# Supplementary material for: Structural basis of p62/SQSTM1 helical filaments and their role in cellular cargo uptake
Source: Nat Commun. 2020 Jan 23;11:440. doi: 10.1038/s41467-020-14343-8 (PMC6978347; doi:10.1038/s41467-020-14343-8)
Supplement: Supplementary file 1 — Supplementary Information [file 41467_2020_14343_MOESM1_ESM.pdf]

Supplementary Information

Structural basis of p62/SQSTM1 helical filaments and their role in cellular cargo uptake

Arjen J. Jakobi et al.

Supplementary Tables

Supplementary Table 1. Primer sequences used in this manuscript.

Primer name	sequence (5' -> 3')
AtNBR1 M1 fw	gcttccatgggcatggagtctactgctaacgcactcg
AtNBR1 K11a fw	gctaacgcactcgtcgtcgccgtgagctatggaggtgtg
AtNBR1 K11A rv	cacacctccatagctcacggcgacgacgagtgcgttagc
AtNBR1 R19A_R20A fw	gtgagctatggaggtgtgcttgcggccttcagggtgcctgttaaagc
AtNBR1 R19A_R20A rv	gctttaacaggcaccctgaaggccgcaagcacacctccatagctcac
AtNBR1 D60A fw	gagtctgacttactctgctgaggatggggatgtgg
AtNBR1 D60A rv	ccacatccccatcctcagcagagtaagtcagactc
AtNBR1 E61A fw	gtctgacttactctgatgcggatggggatgtgg
AtNBR1 E61A rv	ccacatccccatccgcatcagagtaagtcagac
AtNBR1 D60A_D62A fw	gagtctgacttactctgctgaggctggggatgtggttgcccttgt
AtNBR1 D60A_D62A rv	acaagggcaaccacatccccagcctcagcagagtaagtcagactc
AtNBR1 D64A fw	ctgatgaggatggggctgtggttgcccttgt
AtNBR1 D64A rv	acaagggcaaccacaGccccatcctcatcag
AtNBR1 S94 rv	gcttgcggccgcttattaggacacgccagcgttcacattg
TFG M1 fw	gcttccatgggcaacggacagttggatctaagtggg
TFG R95 rv	gcttgcggccgcttattatcttggctggccattaacaaataatg
PKCz G11 fw	gcttccatgggcagcggcggccgcgtccgcc
PKCz E101 rv	gcttgcggccgcttattactcaggggtgctcgggaaaac
NBR1 D50R fw	gatggatacctcttcattttcctcacgcaggtattttatttgaatagtattc
NBR1 D50R rv	gaatactattcaaataaaatacctgcgtgaggaaaatgaagaggtatccatc
p62 M1 fw	gcttccatgggcgcgtcgctcaccgtgaaggc
p62 K102 rv	gcttgcggccgcttattatttctctttaatgtagattcgg
p62 V122 rv	gcttgcggccgcttattacaccatgttgcggggcgcctcc
TFG-P62 fw	tctgttccaggggcccatggccatgaacggacagttggatctaa
TFG-P62 rv	gcagccatcgcagatcacattgggattaacaaataatgtcagtttcagt
TFG-CC-min-pP62 fw	tctgttccaggggcccatggccatgaacggacagttggatctaa
TFG-CC-min-pP62 rv	actccatctgttcctcagggcgtccaggtggttccaagct
MEK5 A5 fw	tttcagggcgccatgggcgcccttggcccctttcct
MEK5 A108 rv	gctcgagtgcggccgctcaggctcttggaaatatctgcag

Supplementary Figures

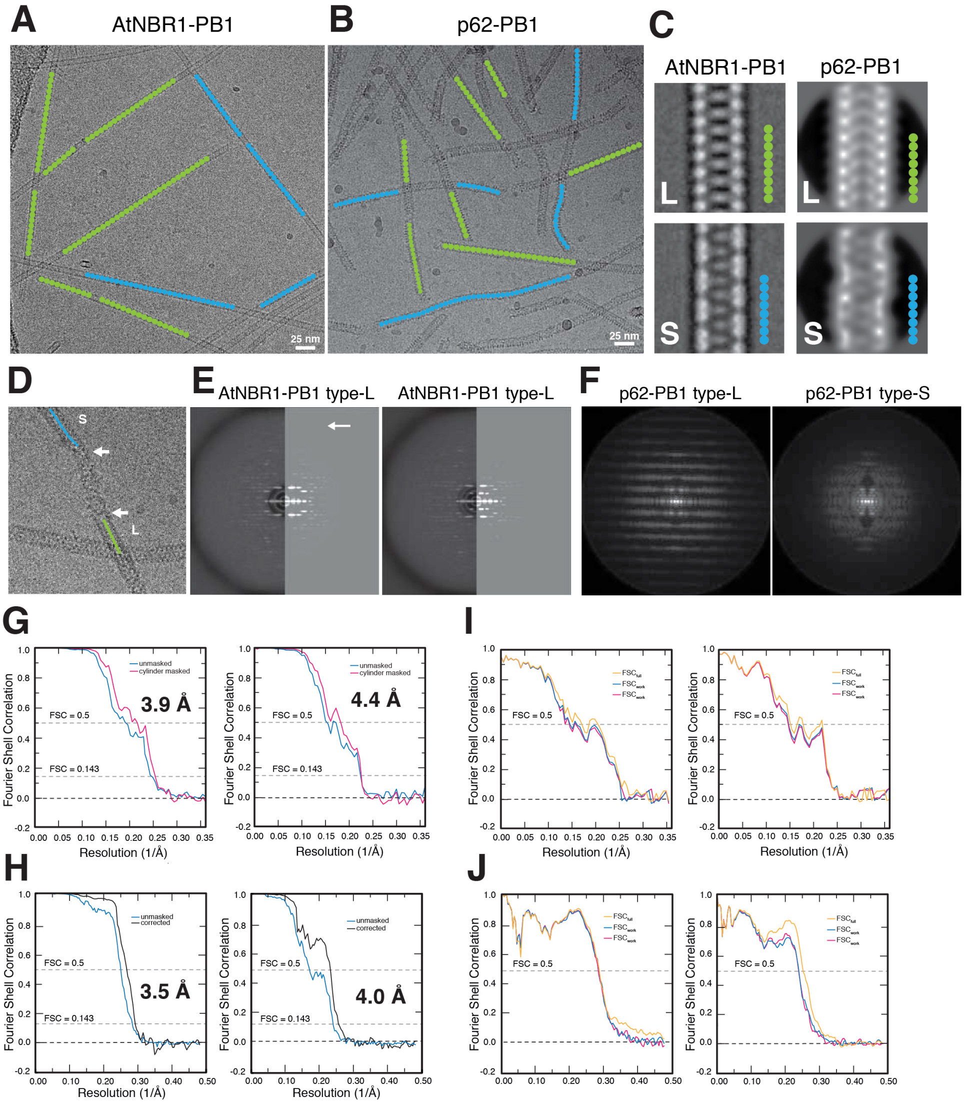

Supplementary Figure 1. Electron cryo-microscopy of AtNBR1-PB1 and p62-PB1.

(A) Representative micrographs of AtNBR1-PB1 with helix traces of segment centers classified as L-type (green) or S-type (blue) superposed. (B) Representative micrographs of p62-PB1 with center traces of segments classified as L-type (green) or S-type (blue) superposed. (C) Low-pass filtered class averages of L-type (top) and S-type (bottom) AtNBR1-PB1 and p62-PB1 assemblies. (D) Representative image showing transitions between L and S-type assemblies for p62-PB1 (E) Side-by-side power spectra of L-type (top) and S-type (bottom) AtNBR1-PB1 assemblies with the power of sum of segments (left) and that simulated from re-projection of the 3D structure (right). Arrows indicate high-resolution meridional layer lines. (F) Power spectra of L-type (top) and S-type (bottom) p62-PB1 assemblies with the summed power spectra of the 2D classes. (G) Fourier shell correlation for 3D reconstruction of L-type (left) and S-type (right) AtNBR1-PB1 assemblies. (H) Fourier shell correlation for 3D reconstruction of L-type (left) and S-type (right) p62-PB1 assemblies. (I) Model vs. map Fourier shell correlation for L-type (left) and S-type (right) AtNBR1-PB1 assemblies. (J) Model vs. map Fourier shell correlation for L-type (left) and S-type (right) p62-PB1 assemblies.

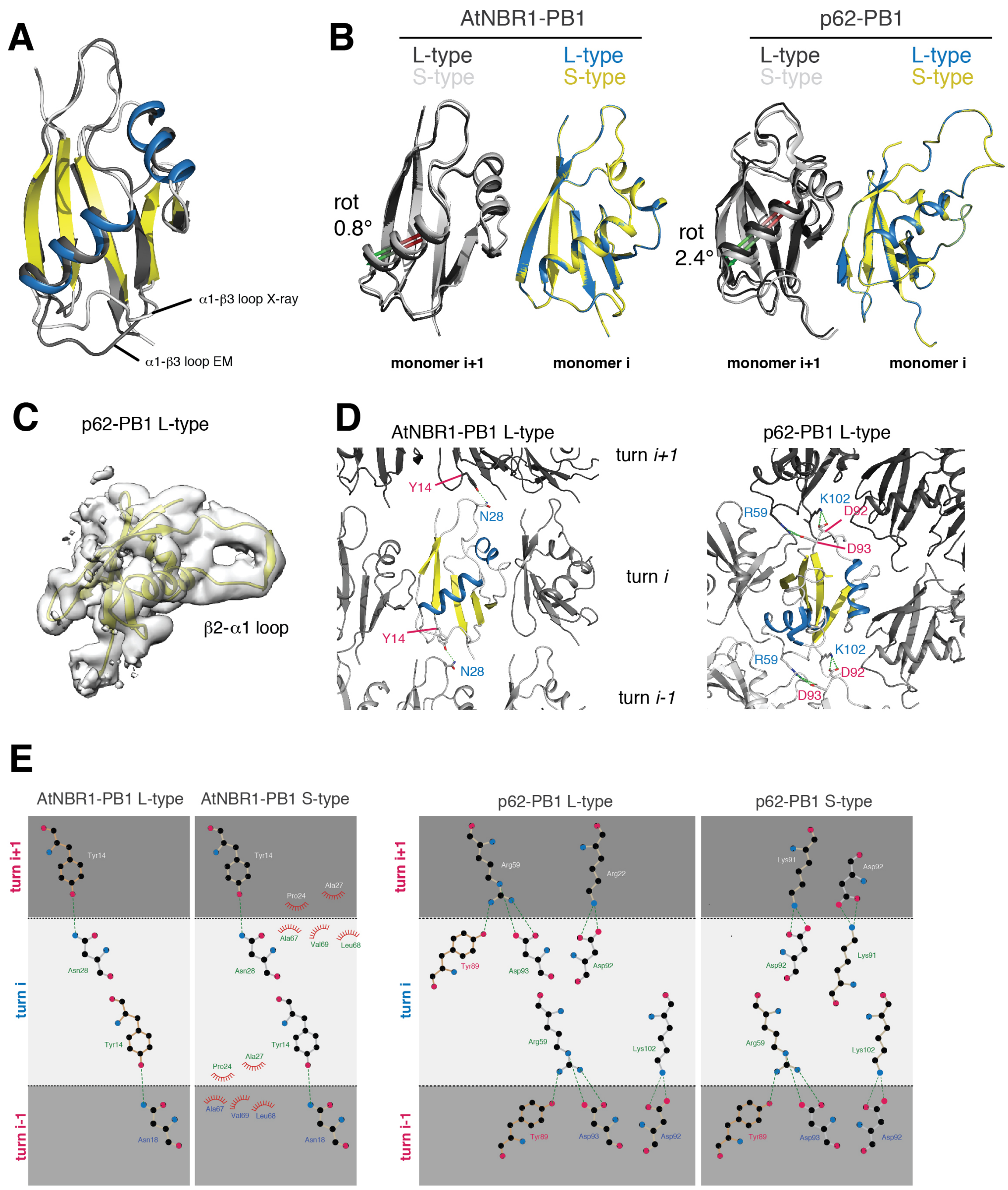

Supplementary Figure 2. Atomic models from crystal and cryo-EM structures of AtNBR1-PB1$^{1\text{-}94}$ and p62-PB1$^{1\text{-}122}$.

(A) Superposition of cartoon representation of atomic models from the 1.6 Å crystal structure of AtNBR1-PB1 and the de novo-built model based on the 3.9 Å cryo-EM AtNBR1-PB1 density map (α-helix: blue, β-strands: yellow). Marked differences are observed in loop regions mediating lateral contacts. (B) Superposition of atomic models for L-type (blue) and S-type (yellow) assemblies for AtNBR1-PB1 (left) and p62-PB1 (right). Monomer *i* for each assembly is superposed and the difference in rotation of adjacent subunit *i+1* are indicated. Only minor differences are observed. (C) LocScale map for L-type p62-PB1 cropped around one monomer. (D) Lateral contacts formed along the helical axis shown for AtNBR1-PB1 (left) and p62-PB1 (right). Subunits are shown in cartoon representation and relevant residue contacts are highlighted with side-chains shown as stick. (E) Schematic representation of common longitudinal contacts formed in AtNBR1-PB1 and p62-PB1 helices.

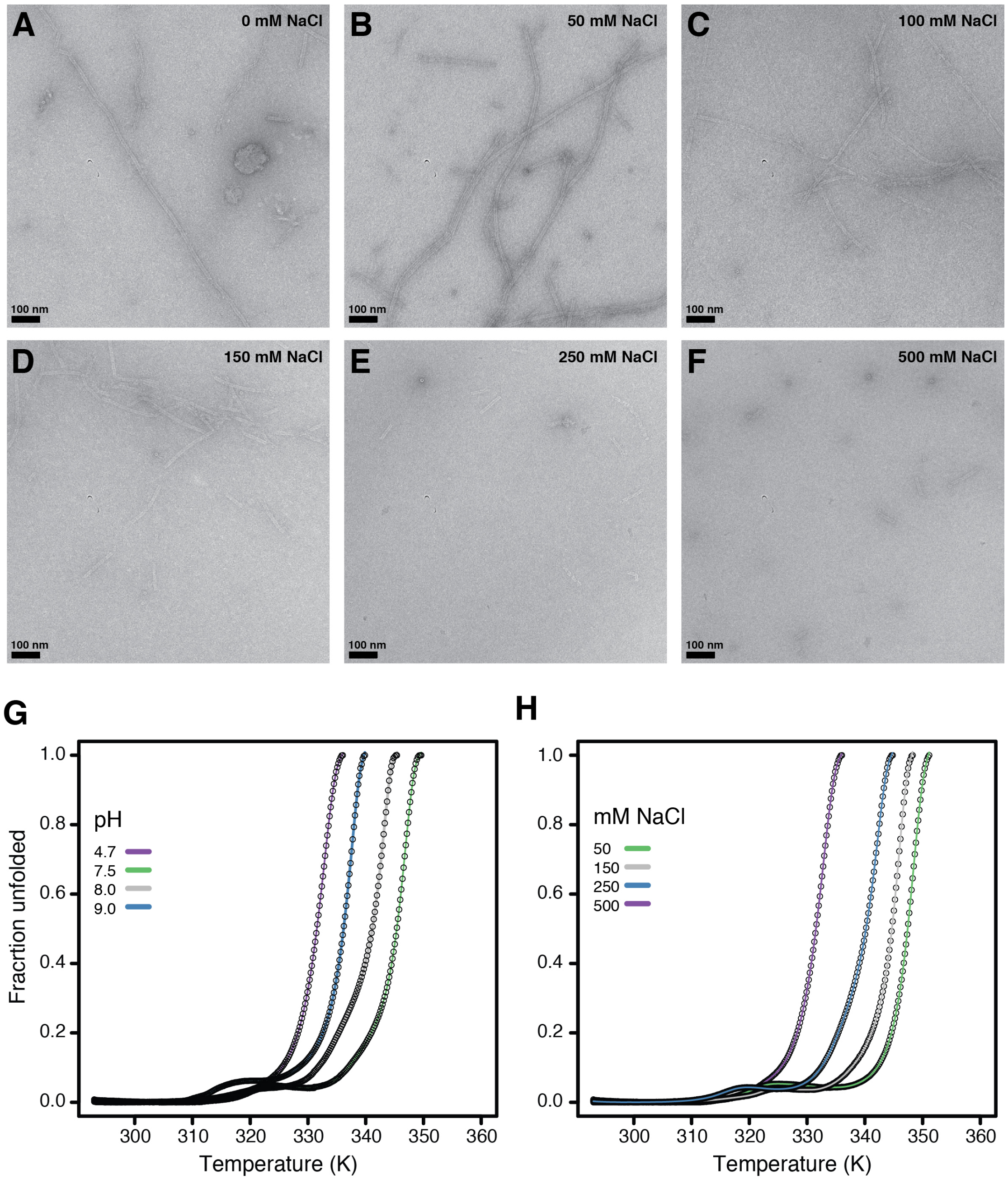

Supplementary Figure 3. Electrostatic interactions drive type PB1 filament formation.

(A-F) Negative-stain electron micrographs of AtNBR1-PB1 at increasing NaCl concentrations (A, 0 mM; B, 50 mM; C, 100 mM; D, 150 mM; E, 250 mM; F, 500 mM) illustrate how ionic strength weakens PB1 homo-oligomerization and affects filament length. (G/H) Thermofluor protein unfolding curves demonstrate that high ionic strength, as well as low and high pH destabilize a thermodynamically favorable (filamentous) state of AtNBR1-PB1.

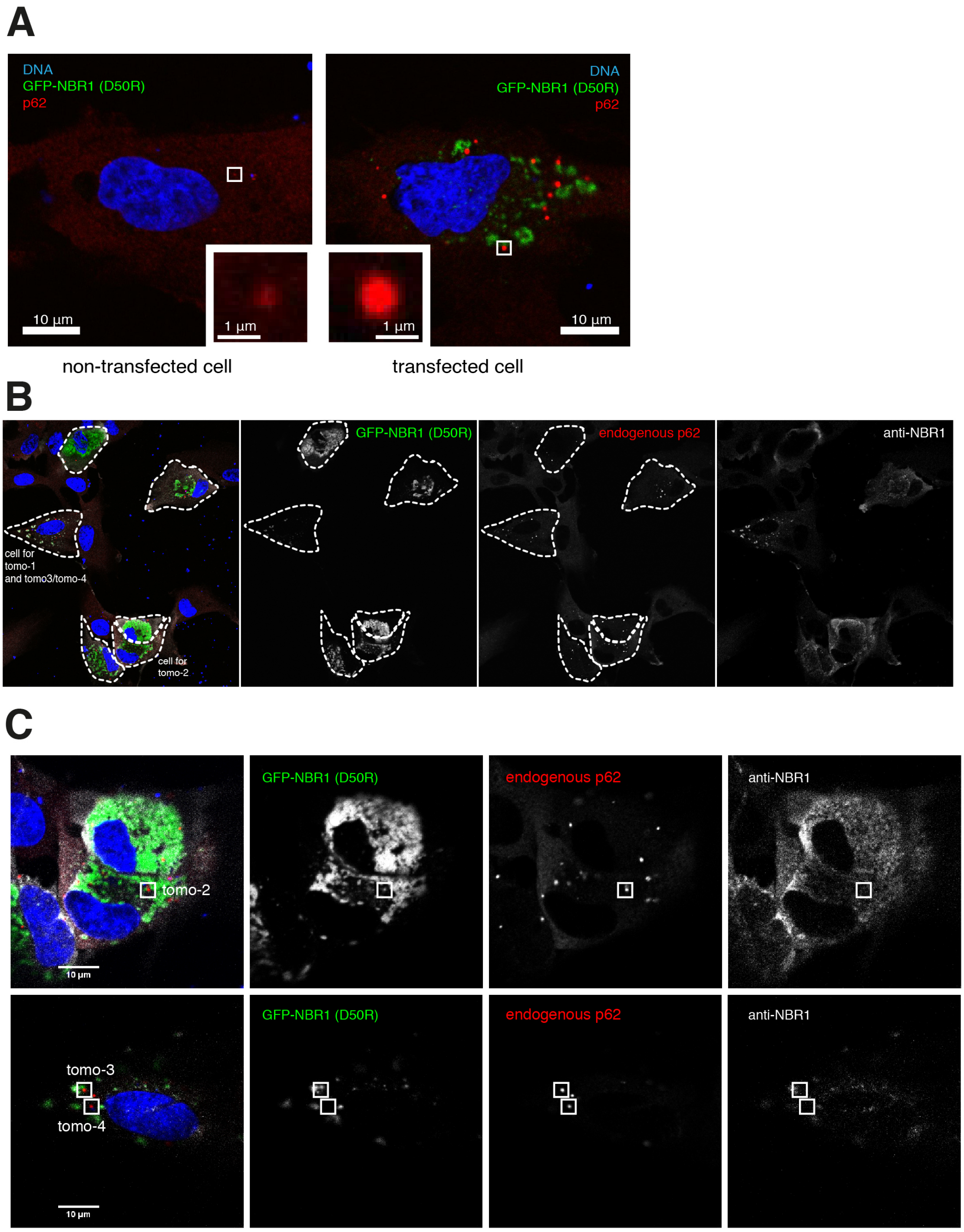

Supplementary Figure 4. Fluorescence images of RPE1 cells.
(A) Representative confocal fluorescence image of RPE1 cells expressing or not expressing NBR1(D50R): DNA (blue), NBR1 (D50R) (green) and endogeneous p62 (red). Note the difference in average dot size of mCherry-p62 observed for both cases. (B) Overview fluorescence image showing the cells used for tomogram acquisition. Cells are outlined and the tomogram number is indicated. (C) Close-up view of cells in (B) indicating the subcellular position for tomogram acquisition.

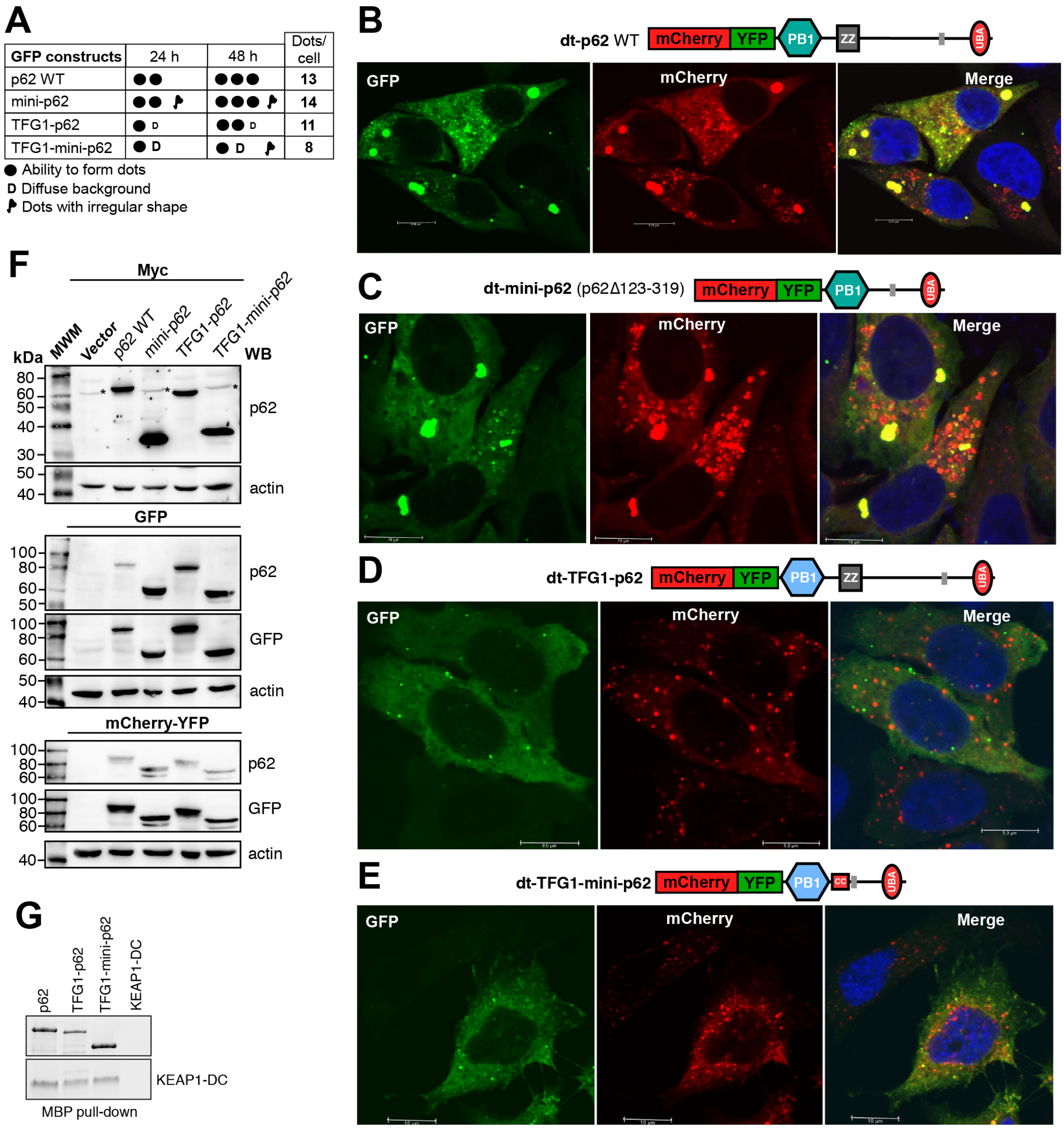

Supplementary Figure 5. Chimera variants of p62 with the PB1 domain exchanged with a related domain from TFG is efficiently degraded by autophagy. (A) Graphic presentation of phenotypes observed by confocal imaging of cells transfected with p62 constructs fused to GFP (number of dots, morphology of dots, and amount of diffuse protein). Cells were analyzed 24h and 48h after transfection. (B-E) Representative confocal images of HeLa p62 KO cells transiently transfected with the indicated p62 constructs fused to the mCherry-YFP double tag. Efficient degradation by autophagy is indicated by the accumulation of red-only dots. Scale bars, 10 μm. (F) Representative western blots using extracts from HeLa cells transiently transfected with the indicated p62 constructs fused to Myc (top), GFP (middle) or mCherry-YFP (bottom). p62, GFP or actin antibodies were used as indicated. Source data are provided as a Source Data file. (G) SDS-PAGE of pull-down fractions for KEAP1-DC using MBP-tagged TFG1-P62 chimera constructs. Source data are provided as a Source Data file.

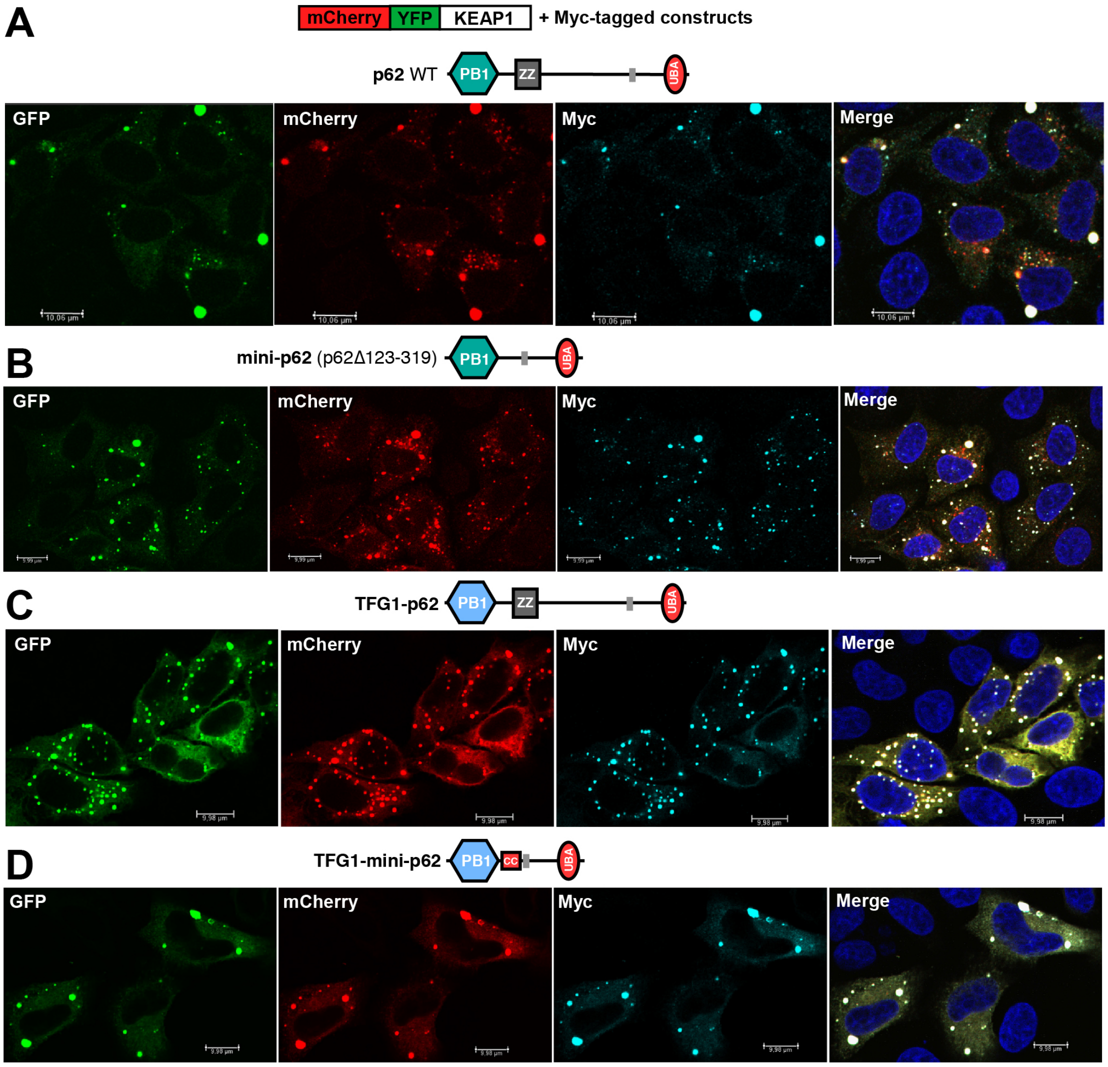

Supplementary Figure 6. p62-mediated degradation of co-expressed KEAP1 depends on the native PB1 domain of p62.

(A-D) Representative confocal images of HeLa p62 KO cells transiently co-transfected with mCherry-YFP tagged KEAP1 and the indicated p62 constructs fused to Myc. Degradation of KEAP1 by autophagy (accumulation of red-only dots) is seen in cells co-transfected with full-length p62 or a mini-p62 deleted for residues 123-319, but not in cells co-transfected with chimera constructs containing the PB1 domain of TFG1. Scale bars, 10 μm.

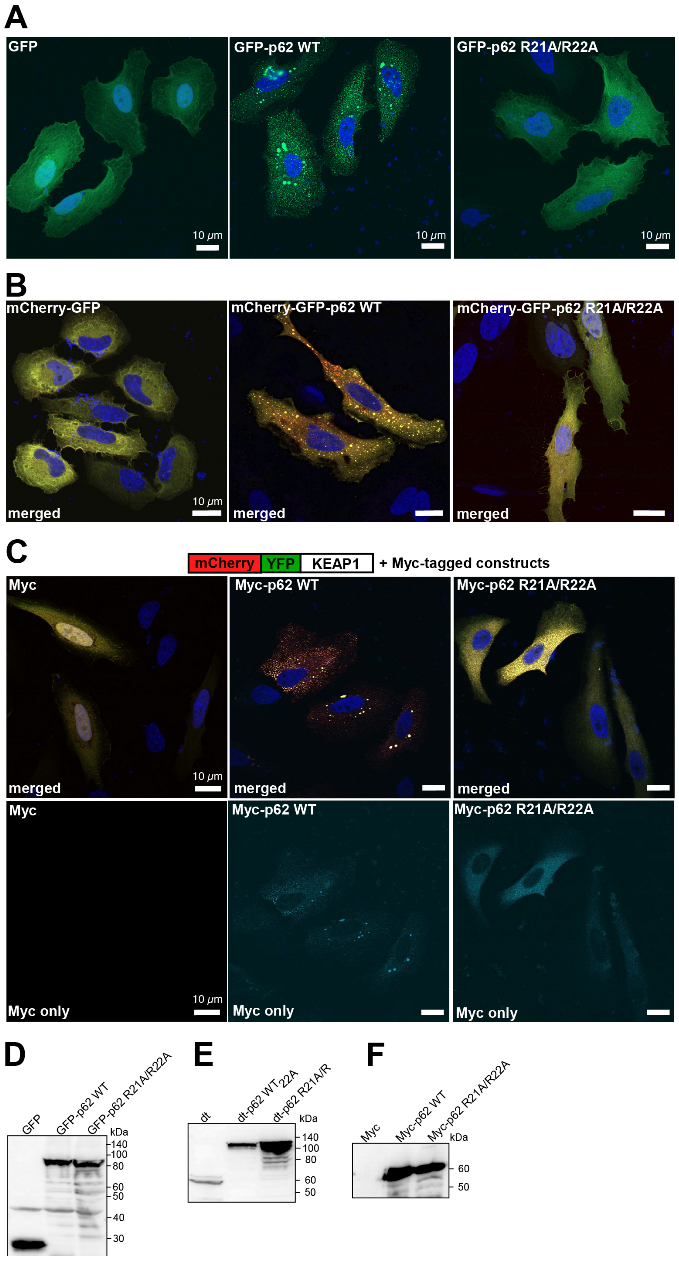

Supplementary Figure 7. The double arginine finger in the PB1 domain is essential for aggregation and autophagy degradation of p62 and its substrate KEAP1.
(A-C) Representative confocal fluorescence images of HeLa p62 (KO) cells expressing GFP only (left), GFP-tagged p62 WT (center) or p62 R21A/R22A (right) (A), and of HeLa p62 (KO) cells expressing mCherry-GFP tagged p62 WT, p62 R21A/R22A or mCherry-GFP alone (B). As seen in (A) the R21A/R22A mutant is unable to form punctate structures and the lack of red dots (B) indicates that this construct is not turned over by autophagy. (C) Representative confocal fluorescence images of HeLa p62 (KO) cells expressing the corresponding myc-tagged p62 WT and p62 R21A/R22A as well as mCherry-YFP-KEAP1. The R21A/R22A mutant does not form aggregates with KEAP1 and is unable to induce lysosomal-localization of mCherry-YFP-KEAP1. Scale bars, 10 μm. (D-F) Western blots of extracts from HeLa cells expressing GFP-tagged (D), mCherry-GFP-tagged (dt) (E) or Myc-tagged (F) p62 WT or p62 R21A/R22A constructs. GFP (D and E) and MYC antibodies (F) were used. Source data are provided as a Source Data file.

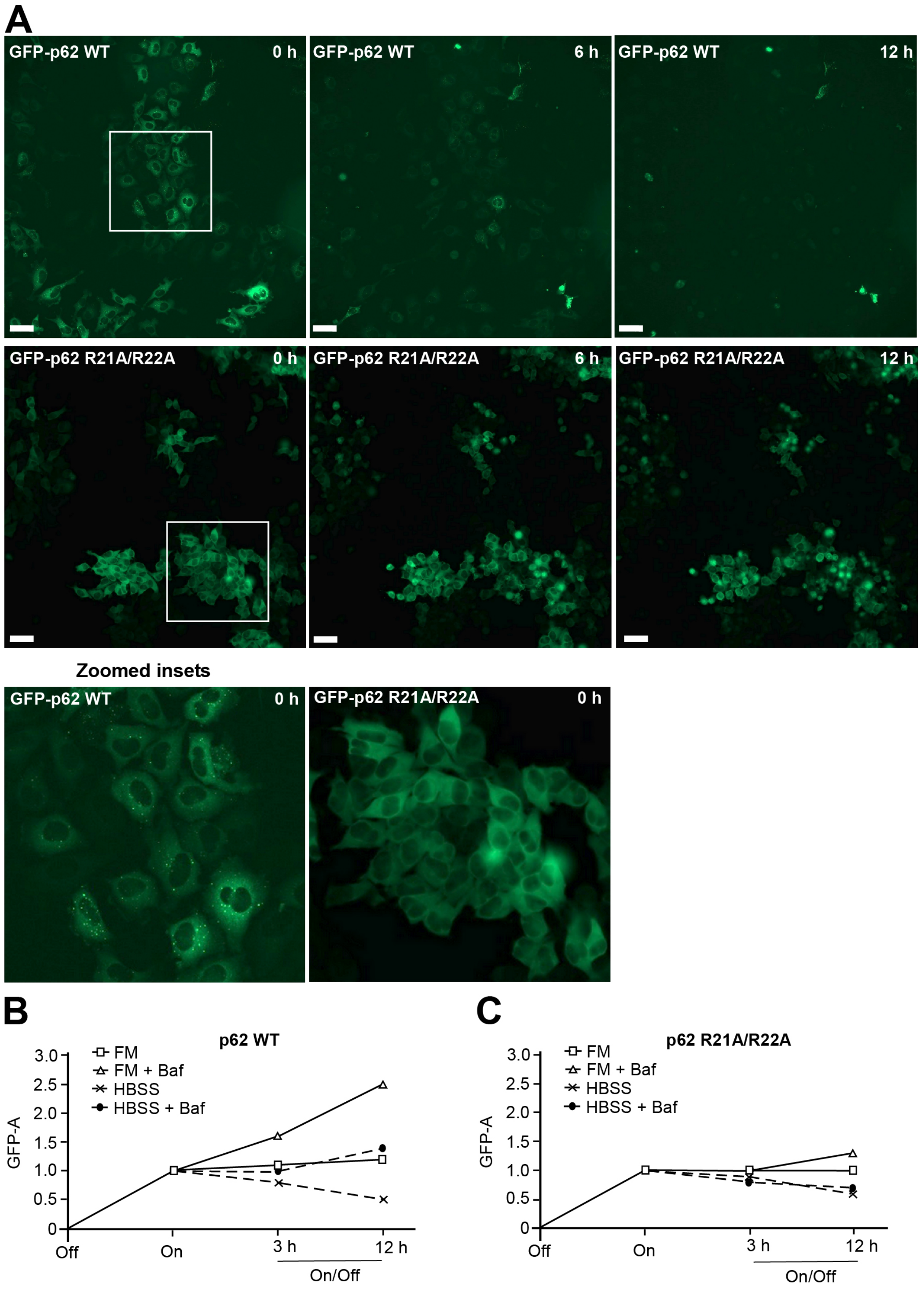

Supplementary Figure 8. The double arginine finger in the PB1 domain is required for autophagy degradation of p62.

(A) Representative still images from movies of HeLa FlpIn T-Rex p62 KO cell lines stably expressing either GFP, GFP-p62 WT or GFP-p62 R21A/R22A at 0 h, 6h and 12h incubation in starvation medium (HBSS). Expression was turned on with tetracycline for 24h before the experiment. Scale bars, 50 μm. Flow cytometry data showing the GFP fluorescence intensity of (B) GFP-p62 WT and (C) GFP-p62 R21A/R22A after 3h or 12h incubation in full medium (FM), FM + Bafilomycin A1 (Baf), HBSS or HBSS + Baf. Cells were untreated (Off) or treated with tetracycline for 24h (On) before the experiment in order to induce the expression of the GFP proteins.
