# Supplementary material for: Structural basis of p62/SQSTM1 helical filaments and their role in cellular cargo uptake
Source: Nat Commun. 2020 Jan 23;11:440. doi: 10.1038/s41467-020-14343-8 (PMC6978347; doi:10.1038/s41467-020-14343-8)
Supplement: Supplementary file 3 — Description of Additional Supplementary Files [file 41467_2020_14343_MOESM3_ESM.pdf]

Description of Additional Supplementary Files

File name: Supplementary Movies 1 and 2

Description: The double arginine finger in the PB1 domain is required for autophagy degradation of p62. The movies show the degradation that occurs during 12h for GFP-p62 WT (Movie 1) and which does not occur for GFP-p62 R21A/R22A (Movie 2) constructs stably expressed in HeLa FlpIn T-Rex p62 (KO) cells. Cells were treated with tetracycline for 24h before the experiment in order to induce the expression of the GFP proteins after which the cells were placed in HBSS to induce starvation-induced autophagy. The movies (4 frames per hour) were obtained using a Zeiss Cell Discoverer 7 with objective 20x0.95 water with fluorescence wavelength 450-488mm. The microscope has an Orca Flash 4.0 Image device.
